# Rehabilitation of the orbital defect post mucormycosis: a case report

**DOI:** 10.1186/s13256-025-05605-4

**Published:** 2025-10-16

**Authors:** Madhavi Selukar, Surekha Godbole, Poorvi Jain, Sharayu Nimonkar, Seema Sathe, Anjali Borle, Abhilasha Bhargava

**Affiliations:** 1 Department of Prosthodontics, Sharad Pawar Dental College & Hospital, Datta Meghe Institute of Higher Education and Research, (Deemed to Be University), Sawangi (Meghe), Wardha, Maharashtra India; 2 Prosthodontics, Wardha, Maharashtra India; 3 Private Clinic, Delhi, India; 4 Department of General Surgery, Acharya Vinobha Bhave Hospital, Datta Meghe Institute of Higher Education and Research, (Deemed to Be University), Sawangi (Meghe), Wardha, Maharashtra India

**Keywords:** Mucourmycosis, Orbital prosthesis, Iris positioning

## Abstract

**Background:**

To restore facial structure and function, prosthodontics plays an essential role in the rehabilitation of individuals with orbital abnormalities. Personalized solutions that promote ocular health and increase quality of life are intended, in addition to improving facial looks. Multidisciplinary collaboration between ophthalmologists, prosthodontists, maxillofacial surgeons, and other experts is often necessary to provide comprehensive care that addresses both the physical and psychological well-being of the patient. Devastating orbital abnormalities such as tissue necrosis, loss of structural integrity, and possibly vision impairment can result from this. A multidisciplinary approach is necessary to address the challenging orbital abnormalities associated with mucormycosis.

**Case report:**

A 54-year-old Asian female patient, resident of the Vidarbha region of Maharashtra, India, presented to the outpatient department of Prosthodontics and Crown and Bridge with a chief complaint of a missing left eye. The patient’s previous medical history revealed that she was operated for mucormycosis post coronavirus disease 2019. Prosthodontic rehabilitation of the missing left eye was done, and the orbital defect was rehabilitated.

**Conclusion:**

Healthcare professionals must comprehend the connection between ocular abnormalities and mucormycosis to guarantee prompt diagnosis, efficient therapy, and better patient outcomes in this difficult clinical situation. Advanced techniques and materials in prosthodontic rehabilitation aim to deliver durable results that restore functionality and rebuild patients’ confidence.

## Background

Patients with orbital deformities may suffer greatly not only from a functional impairment of vision but also from a noticeable cosmetic abnormality. Since vision cannot be restored, significant progress has been made in the last 10–20 years to enhance the cosmetic rehabilitation of orbital injuries. Surgical procedures, congenital malformations, trauma, oncological resections, and other reasons can result in orbital defects, which are defined as structural abnormalities or losses in the orbital region. These flaws can have a major effect on appearance as well as functionality, which can cause problems with eyesight, face symmetry, and self-esteem in general [1].

Mucormycosis, also known as zygomycosis, is a rare but serious fungal infection caused by a group of molds called mucormycetes. These molds are commonly found in the environment, particularly in soil, decaying wood, and organic matter. It is characterized by tissue necrosis due to an invasion of blood vessels and subsequent thrombosis, which usually follows a rapid progression. When it affects the orbital region, mucormycosis, a dangerous fungal illness brought on by molds in the Mucoraceae family, can cause serious consequences. People with weakened immune systems, such as those with uncontrolled diabetes, cancer, or undergoing immunosuppressive therapy, are more likely to get an opportunistic infection. When sinuses are affected by mucormycosis, the infection can move quickly to the orbit and adjacent structures, resulting in rhinocerebral mucormycosis [2].

Rehabiliting patients with orbital defects presents a challenge and requires meticulous surgical and prosthetic treatment planning to attain the desired outcome. This case report provides a clear understanding of the surgical and prosthetic phase of mucourmycosis post coronavirus disease (COVID) infection and addresses the challenges perceived during the prosthetic rehabilitation.

## Case report

A 54-year-old female, resident of Vidarbha region of Maharashtra, India, presented to the OPD of the Department of Prosthodontics and Crown and Bridge with a complaint of a missing left eye. The past medical history of the patient revealed that enucleation and exentration of her left eye was done 5 months previously, secondary to mucormycosis post COVID infection. The patient had been receiving medications for type 2 diabetes mellitus since 6 years previously.

**Fig. 1:**
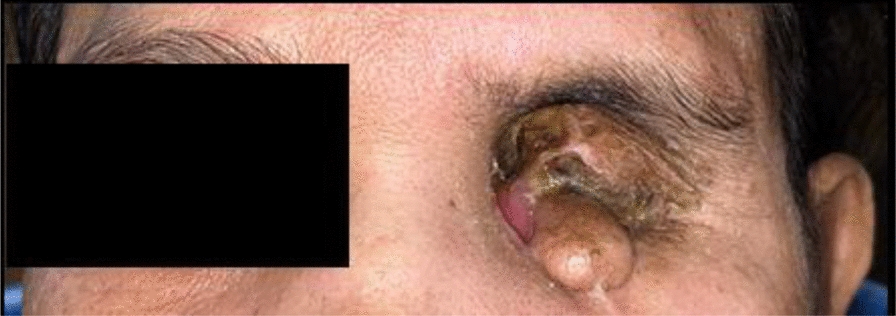
Exenteration of left eye along with soft tissue growth

On inspection, exenteration of the left eye was seen along with a watery discharge and soft tissue growth, as shown in Fig. 1. On palpation, the dimensions of the soft tissue growth were 1.8 × 2.0 cm^2^. The size of the defect was 3.8 cm mediolaterally and 3.1 cm supero-inferiorly. The depth of the defect was –2.8 cm. The defect was nontender on palpation. The defect area over which the prosthesis was planned was not ideal and required further surgical modifications; however, as the patient was not willing to undergo the subsequent surgery, we continued with the prosthetic treatment planning.

### The pre-prosthetic treatment phase


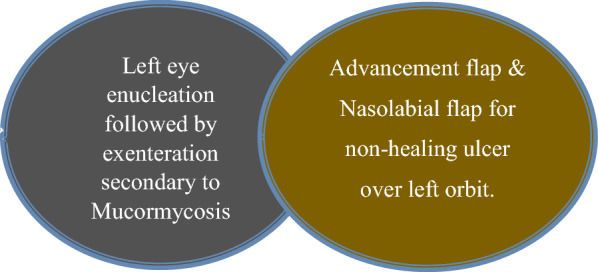


**Fig. 2:**
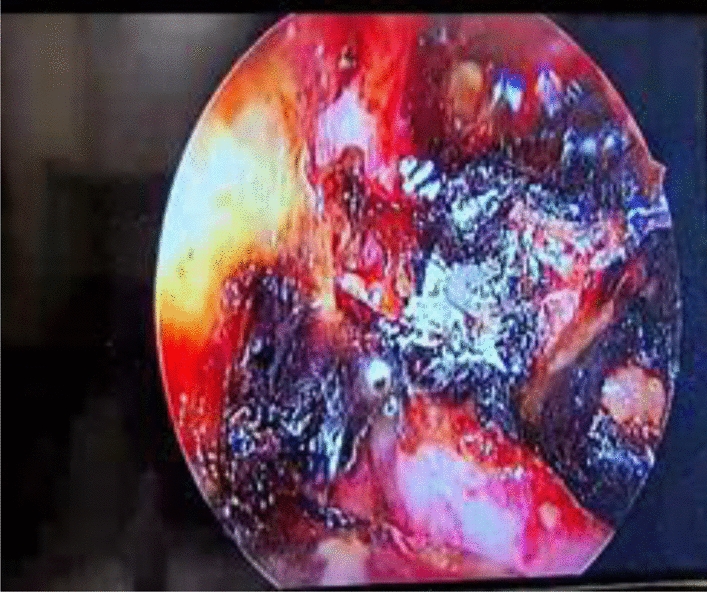
Pre-operative diagnostic nasal endoscopy suggestive of black crust present over maxillary, ethamoid region

At 3 weeks post COVID infection, the patient reported to the Ear, Nose, and Throat (ENT) department with a complaint of left-sided nasal obstruction, left eye pain, and loss of vision with the left eye. Physical examination revealed left ethmoidal sinus tenderness, deviated nasal septum to left, and black debris in left nasal cavity. Pre-operative diagnostic nasal endoscopy was suggestive of deviated nasal septum to left with spur on left. Black crust was present over maxillary, ethmoid region, as shown in Fig. 2.

On contrast enhanced computed tomography of the paranasal sinuses (CECT PNS) with orbit, invasive rhinosinusitis involving left maxillary, ethmoid, bilateral sphenoid, and frontal sinus was seen. Bony involvement and extension into extra- and intracranial compartment of left orbit causing orbital proptosis was suggestive of mucormycosis.

Magnetic resonance imaging (MRI) of brain with orbit was suggestive of (s/o) of left-sided pansinusitis with left orbital involvement and extension.

On the basis of the above investigations, the final diagnosis was post-COVID left pansinusitis with left orbital cellulitis of fungal origin.

Considering this diagnosis, surgery was planned: left endoscopic sinus surgery and left orbital exenteration by ophthalmologist.

### Surgical phase

**Fig. 3:**
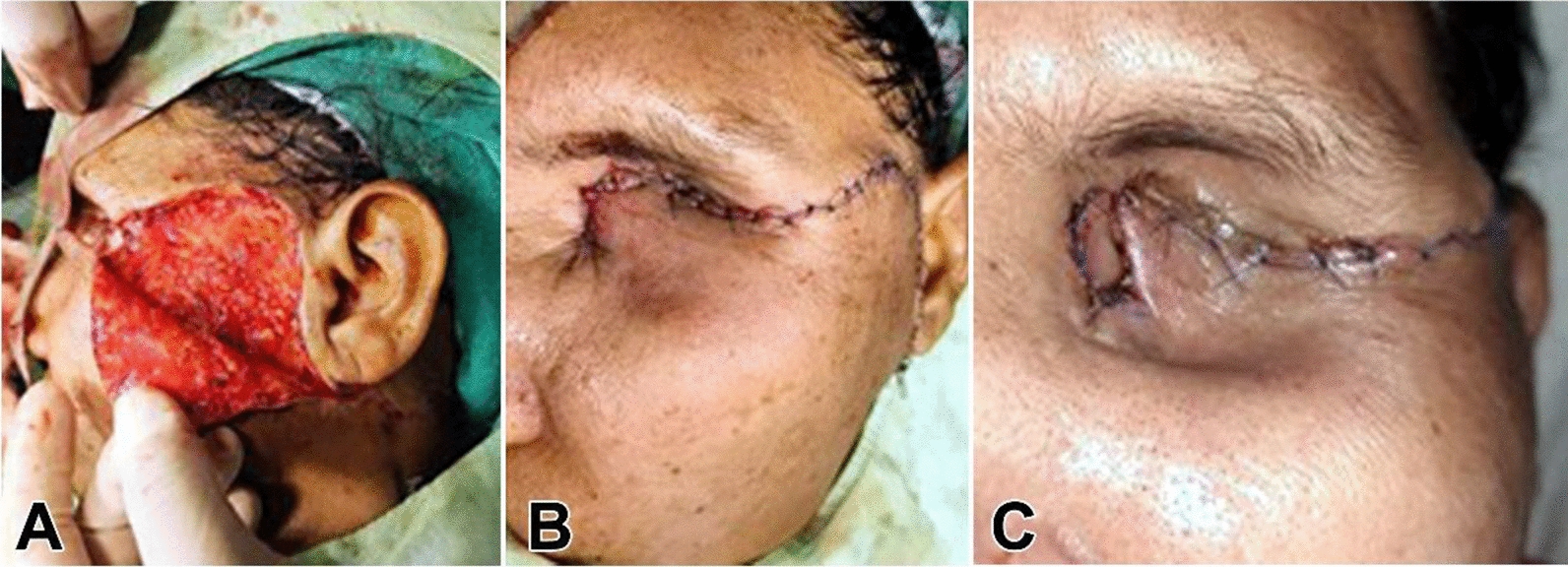
Reflection of cheek flap and positioning

Under general anesthesia (GA), endoscopic sinus surgery was performed; all sinuses were opened anteriorly to posteriorly: maxillary sinus, anterior and posterior ethmoidal sinus, and sphenoid sinus. Yellowish-green mucopurulent discharge was present in all sinuses and was suctioned. Dead necrotic discharge was present in sphenoid sinus and removed, thus meticulous cleaning of all the sinuses was done and sent for KOH staining and histopathological examination (HPE); this was followed by left orbital exenteration by ophthalmologist. Before proceeding with the surgical intervention, it was ensured that there was no residual mucormycosis involved in the nonhealing ulcer over the left orbit. After pre-operative assessments, the patient was planned for a cheek flap to cover the ulcer. Ulcer edges were excised, and a cheek flap was advanced into the defect and placed without tension, as shown in Fig. 3a, b, c. After 3 weeks, upper and medial part of suture site gave way to an ulcer. Since the vascularity was intact, the blood sugar levels of the patient were uncontrolled with recurrence of ulcer. After reassurance of no residual pathology by the ENT Department, the patient was scheduled for advancement of nasolabial flap.

A left nasolabial flap based superiorly was raised, where the inferior edge of the flap was put into the defect without tension and the donor defect was closed primarily. ENT consideration and postoperative follow-up of the patient were observed.

### Prosthodontic management

**Fig. 4:**
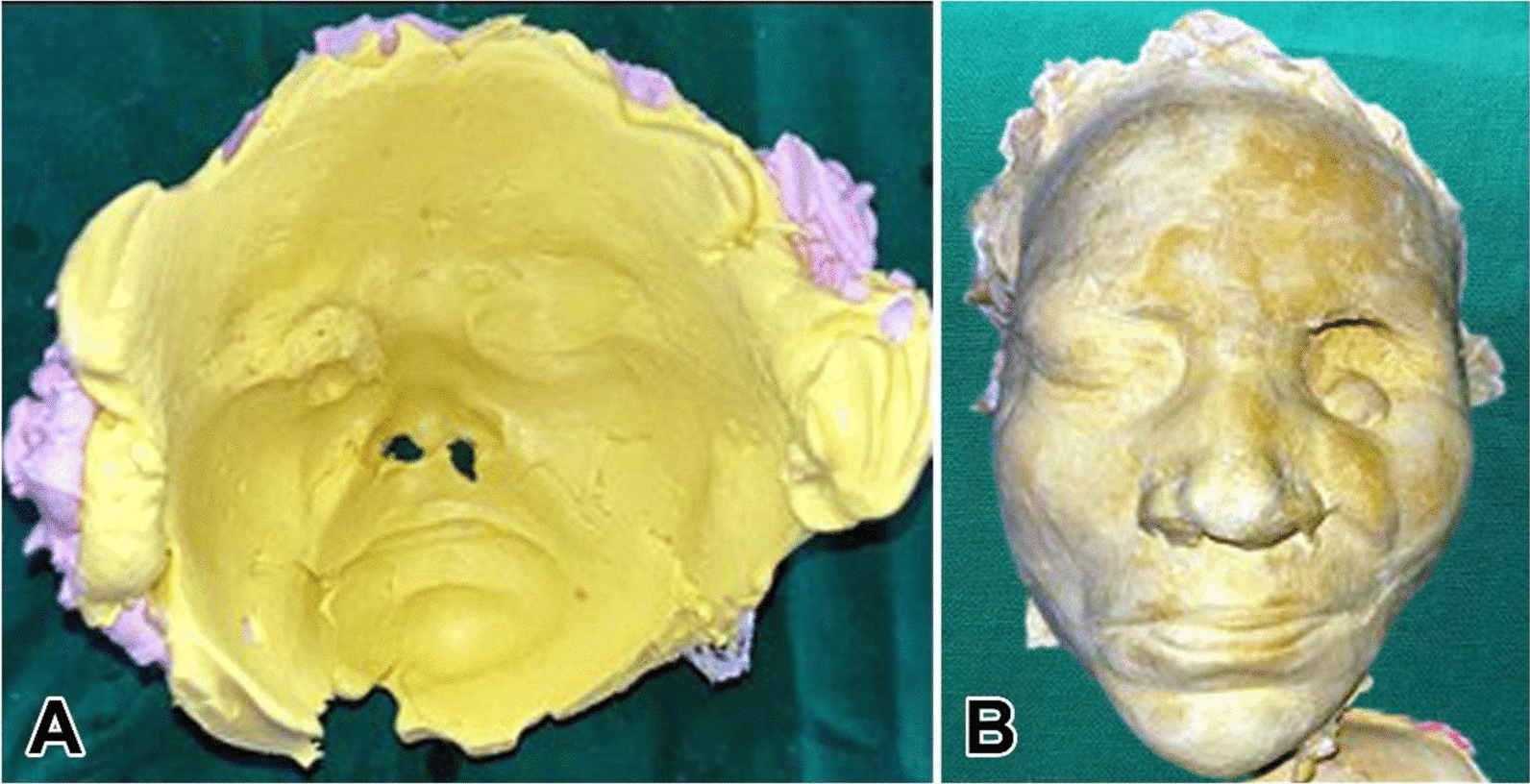
Depict facial moulage

**Fig. 5:**
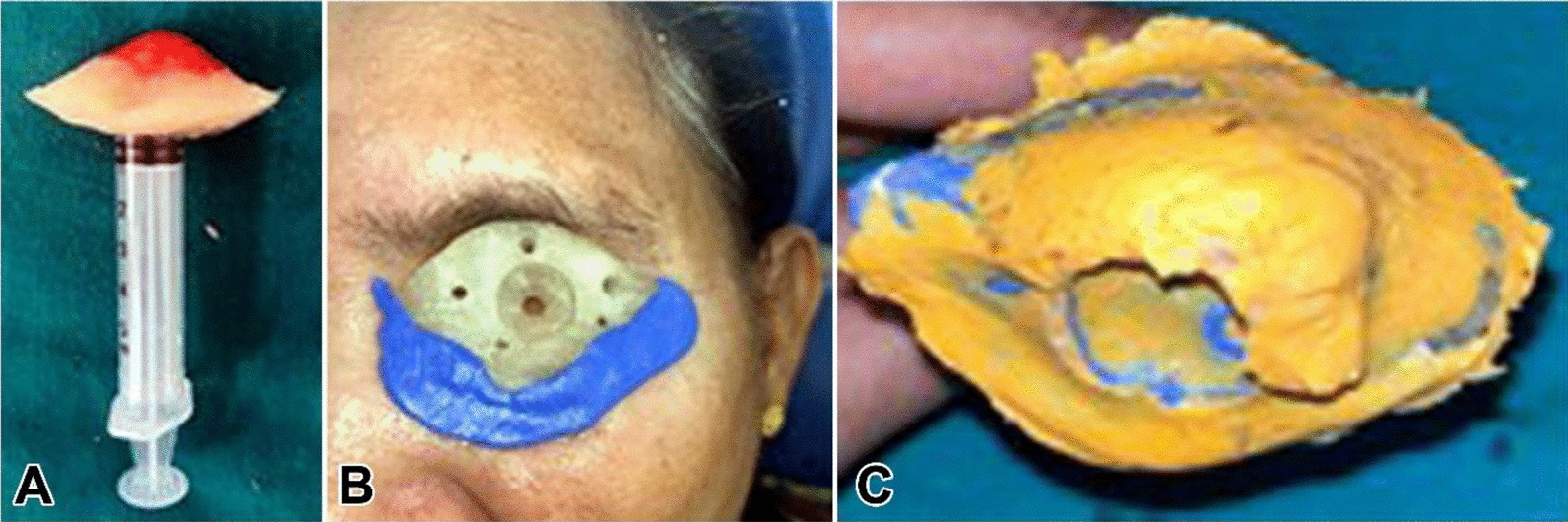
Depiction of the steps in recording the final impression: **a**, **b** checking the adaptation of custom tray and tray modification and **c** the final impression of the orbital defect

A conventional orbital prosthesis was planned. Even when the patient is not present, a correct facial moulage aids in understanding the orientation and appropriate placement of the face prosthesis in relation to other facial landmarks. Elastomeric impression material, alginate, and dental plaster were applied directly over the patient’s face to create an impression for the purpose of constructing a facial moulage, according to methods previously published in literature. Taking a preliminary impression is the initial step in the prosthetic fabrication process, as shown in Fig. 4a and b. The purpose of making this impression is to help in creating a working model that will guide the design and fit of the orbital prosthesis. This impression is often used to create a custom tray for the definitive impression [3]. An orbital prosthesis is made using a secondary impression, which offers more precision than the preliminary impression. For improved detail capture, one usually makes use of polymers such as polyvinyl siloxane, as shown in Fig. 5a, b, c. To guarantee a tight fit, a customized tray is often made using the initial impression. All pertinent contours are captured by applying and setting the impression material over the anatomical region. An accurate functional model for the finished prosthesis is then made using this comprehensive imprint [4, 5].

### Selection of eye shells, shade matching, and wax pattern fabrication

Achieving a natural appearance with an orbital prosthesis requires careful consideration of shade selection. Using a shade guide under natural lighting, the prosthesis is colored to match the patient’s skin tone. During the choosing process, factors including age, ethnicity, and surrounding hues must be taken into account. Involving the patient in this process helps guarantee that they will be happy with the finished appearance [7].

One starts with an exact model made from the secondary impression while creating a wax pattern. To ensure symmetry and proportion, the wax is layered to mimic the anatomical outlines of the missing orbital component. To achieve realism, refinement is essential; the wax pattern must be smoothed and detailed [8].

**Fig. 6:**
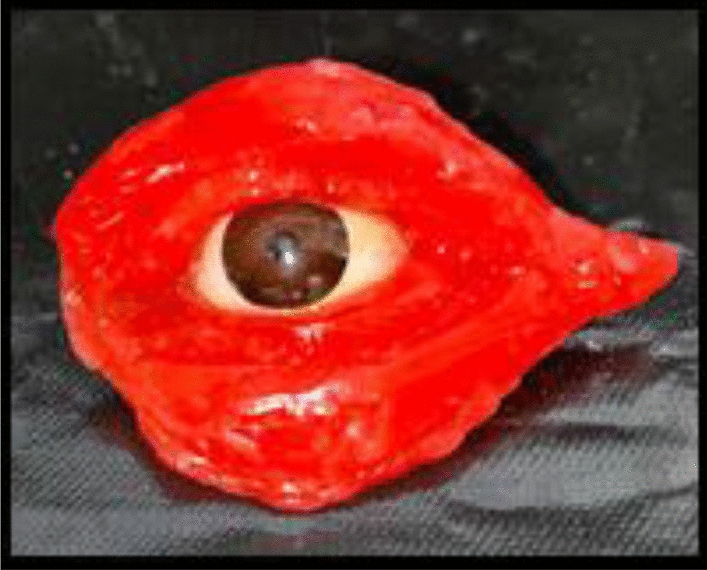
Wax pattern fabrication

Wax pattern fabrication was done as shown in Fig. 6 followed by iris positioning. To guarantee an even distribution and get rid of air bubbles, the chosen prosthetic material—such as silicone or acrylic—is carefully poured into the mold to start the packing procedure for an orbital prosthesis. To ensure appropriate hardness, the material is packed and allowed to cure in accordance with the manufacturer’s specifications. The prosthesis is carefully demolded after it has cured, being careful not to damage it in the process.

After that, flash material is cut away, and the edges are smoothed out to blend in with the surrounding anatomy’s curves for a more realistic appearance. Fine abrasives are used to polish the surface of the prosthesis, improving its comfort and appearance. Following trimming, polishing creates a high-gloss finish that enhances the overall appearance of the prosthesis. Additionally, its smooth surface is helpful for upkeep and cleaning [6, 7].

**Fig. 7:**
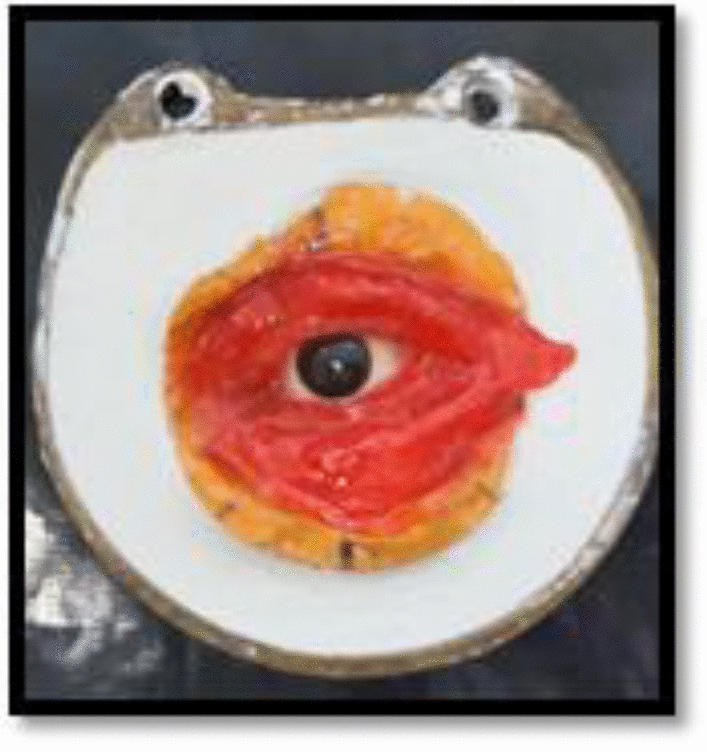
Flasking procedure

Figure 7 explains the flasking procedure of the orbital prosthesis.

To achieve a smooth integration of the prosthesis with the patient’s features, some final stages are essential to ensure that the end product is both esthetically pleasing and useful through careful packing, finishing, and polishing. In the end, these procedures help the patient who is wearing the orbital prosthetic feel more at ease and satisfied [8].

## Discussion

Ocular prostheses are fit behind the eyelids over a shrunken eyeball. An orbital prosthesis restores the eyeball and eyelids and may include the eyebrow and part of the forehead, nose, or cheek [9].

### Classification of orbital defects

Defects are categorized into type 1 (simple orbital exenteration with an intact bony orbit), type 2a (orbital exenteration and removal of a single orbital wall), type 2b (removal of more than one orbital wall), type 3 (orbital exenterations with skull base defects), and type 4 (extended exenterations with penetrating orbitomaxillary defects).

Couto *et al.* (2017) presented a comprehensive methodology for the custom design and manufacture of a soft-tissue prosthesis, focusing on an orbital case study. This approach integrated three-dimensional (3D) scanning, computer-aided design (CAD) modeling, and additive manufacturing to achieve a patient-specific fit and high esthetic outcome. The workflow focuses on facial scanning, followed by digital reconstruction and mold fabrication for silicone casting. The study highlighted the advantages of digital tools in improving precision, reducing production time, and enhancing patient satisfaction. This article demonstrates the feasibility and effectiveness of this technology-driven method in craniofacial prosthetic rehabilitation [10].

### Recent advances in the field of orbital prosthesis

Extra-oral implants in reconstruction of craniofacial defects, achieving proper retention of prosthesis, has become more promising. Implant-supported prostheses may minimize or eliminate the problems associated with conventional prostheses. Enhancing biocompatibility, functioning, and esthetics for patients undergoing evisceration or enucleation has been the focus of recent advancements in orbital implants. The following are some significant developments: Bioactive coatings reduce rejection rates and improve stability, with coatings that encourage tissue integration and cell proliferation [10, 11]; Customization through the use of 3D-printing technology can create implants that are suited to each patient’s unique anatomical features, resulting in a better fit and improved comfort [12, 13]; Less invasive surgical techniques and sophisticated imaging systems have shortened recovery periods and increased implant placement accuracy [14, 15]; Regarding materials, biomaterial innovations that improve integration with neighboring tissues and reduce difficulties include hydrogels and porous polyethylene [16]; A few recent models of smart implants have sensors built in to track healing and identify issues, giving surgeons access to data in real time.

## Conclusion

The loss of an eye through trauma/infection or for any reason is devastating to the patient. Recent advances in the field of maxillofacial prosthesis is opening new horizons and challenging research along the path of advancements in the field of maxillofacial prosthetic rehabilitation of the patient.

## Data Availability

Data are provided within the manuscript.

## Abbreviations

OPD: Outpatient department
s/o: Suggestive of
CECT PNS: Contrast-enhanced computed tomography of the para nasal sinus
MRI: Magnetic resonance imaging

## References

[1] Kusum CK, Indrajeet, Wankhade BG. A simple technique to fabricate a facial moulage with a prefabricated acrylic stock tray: a clinical innovation. J Indian Prosthodontic Soc. 2014;14:341–4. 10.1007/s13191-014-0375-x PMC4502014 26199544

[2] Gamaletsou MN, Sipsas NV, Roilides E, Walsh TJ. Rhino-orbital-cerebral mucormycosis. Curr Infect Dis Rep. 2012;14(4):423–34. 22684277 10.1007/s11908-012-0272-6

[3] Kathuria N, Prasad R, Gupta N, Gulati M, Bhide SV. A modified technique and simplified laboratory procedure for Ocular Prosthesis Fabrication. J Prosthodont Res. 2012;56(2):147–50. 22104621 10.1016/j.jpor.2011.07.002

[4] Doshi PJ, Aruna B. Prosthetic management of patient with ocular defect. J Indian Prosthodontic Soc. 2005;5(1):37–8.

[5] Chalian VA, Drane JB, Standish SM. Maxillofacial prosthetics: multidisciplinary practice. (No Title). 1972.

[6] Manvi S, Ghadiali B. Prosthetic rehabilitation of a patient with an orbital defect using a simplified approach. J Indian Prosthodontic Soc. 2008;8(2):116–8. 10.1007/s13191-012-0128-7 PMC3416935 23997470

[7] Sathe S, Pisulkar S, Nimonkar SV, Belkhode V, Borle A. Positioning of iris in an ocular prosthesis: a systematic review. J Indian Prosthodont Soc. 2020;20(4):345. 33487961 10.4103/jips.jips_374_19 PMC7814693

[8] Lapointe J, Boisvert J, Kashyap R. Next generation artificial eyes with dynamic iris. Int J Ophthalmol Clin Res. 2016;3(3):1.

[9] Eshghpour M, Kheirandish M. Patient satisfaction in maxillofacial prosthetics: a systematic review. Prosthodontics Int. 2022;20(1):30–45.

[10] Couto M, Machado M, Reis A, Neto R, Alves JL. Comprehensive methodology for custom-design and manufacture of soft-tissue prosthesis: orbital case-study. In 2017 IEEE 5th Portuguese meeting on bioengineering (ENBENG). IEEE. 2017. pp. 1–4

[11] Dahlan A, Iradani PM. Custom made orbital prosthesis using digital approach: a case report. World J Adv Res Rev. 2024;23(1):997–1001.

[12] Mazzocca A, Cicciù M. 3D printing in maxillofacial prosthetics: a review. J Prosthet Dent. 2021;126(2):189–97.

[13] Bansod AV, Pisulkar SK, Beri A, Jain R, Deshmukh S, Umre U. Innovative hybrid approach: digital and analog fabrication of orbital prosthesis for post-COVID-19 mucormycosis defects using photogrammetry technique. Sci Rep. 2024;14(1):26446. 39488567 10.1038/s41598-024-77836-2 PMC11531480

[14] Zhang Y, *et al*. Application of CAD/CAM technology in the fabrication of maxillofacial prostheses. Int J Oral Sci. 2022;14(1):10.35153297

[15] Kim HJ, Kim HJ. Recent advancements in silicone materials for maxillofacial prostheses. J Biomed Mater Res. 2023;111(5):765–72.

[16] Jang HW, *et al*. The role of imaging in maxillofacial prosthetics. Clin Oral Investig. 2021;25(9):5043–50.

